# A chromosome-level genome assembly for the paramylon-producing microalga *Euglena gracilis*

**DOI:** 10.1038/s41597-024-03404-y

**Published:** 2024-07-16

**Authors:** Zixi Chen, Yang Dong, Shengchang Duan, Jiayi He, Huan Qin, Chao Bian, Zhenfan Chen, Chenchen Liu, Chao Zheng, Ming Du, Rao Yao, Chao Li, Panpan Jiang, Yun Wang, Shuangfei Li, Ning Xie, Ying Xu, Qiong Shi, Zhangli Hu, Anping Lei, Liqing Zhao, Jiangxin Wang

**Affiliations:** 1 Shenzhen Key Laboratory of Marine Bioresource and Eco-environmental Science, Shenzhen Engineering Laboratory for Marine Algal Biotechnology, Guangdong Provincial Key Laboratory for Plant Epigenetics, College of Life Sciences and Oceanography, Shenzhen University, Shenzhen, 518060 China; 2 State Key Laboratory for Conservation and Utilization of Bio-Resources in Yunnan, Yunnan Agricultural University, Kunming, 650201 China; 3 Yunnan Research Institute for Local Plateau Agriculture and Industry, Kunming, 650201 China; 4 Shenzhen Rare Disease Engineering Research Center of Metabolomics in Precision Medicine, Shenzhen Aone Medical Laboratory Co, Ltd, Shenzhen, 518000 China; 5 College of Chemistry and Environmental Engineering, Shenzhen University, Shenzhen, 518060 China

**Keywords:** Genome, Plant genetics, Water microbiology

## Abstract

*Euglena gracilis* (*E. gracilis*), pivotal in the study of photosynthesis, endosymbiosis, and chloroplast development, is also an industrial microalga for paramylon production. Despite its importance, *E. gracilis* genome exploration faces challenges due to its intricate nature. In this study, we achieved a chromosome-level *de novo* assembly (2.37 Gb) using Illumina, PacBio, Bionano, and Hi-C data. The assembly exhibited a contig N50 of 619 Kb and scaffold N50 of 1.12 Mb, indicating superior continuity. Approximately 99.83% of the genome was anchored to 46 chromosomes, revealing structural insights. Repetitive elements constituted 58.84% of the sequences. Functional annotations were assigned to 39,362 proteins, enhancing interpretative power. BUSCO analysis confirmed assembly completeness at 80.39%. This first high-quality *E. gracilis* genome offers insights for genetics and genomics studies, overcoming previous limitations. The impact extends to academic and industrial research, providing a foundational resource.

## Background & Summary

*Euglena*, a genus of single-celled flagellate eukaryotes, is ubiquitously distributed in both freshwater and saltwater environments. Possessing photosynthetic chloroplasts, *Euglena* exhibits autotrophic characteristics akin to plants, while also displaying heterotrophic attributes similar to animals^1–3^. *E. gracilis*, a prominent species within the genus, serves as a widely utilized model organism in both academic and industrial research due to its rich array of valuable compounds, including pigments, unsaturated fatty acids, vitamins, amino acids, and the distinctive β-1,3-glucan, paramylon—an advantageous functional food ingredient^4–6^. Notably, recent studies, such as Wu *et al*.’s pilot-scale fermentation achieving maximal biomass and paramylon content^7^, underscore the industrial potential of *E. gracilis*.

Despite substantial advancements in genetic modification^8–13^, hindered by the absence of a high-quality genome, *E. gracilis* remains a subject of limited genetic engineering tools and applications. In 2019, Ebenezer *et al*. presented an initial genome assembly of *E. gracilis* (1.43 Gb), which, though informative, proved significantly fragmented^14^. Consequently, researchers have resorted to omics approaches, including *de novo* transcriptome assembly^14,15^ and proteomic analysis^1,14^, to explore physiological and genomic aspects. Nevertheless, a definitive high-quality genome assembly remains a critical prerequisite for advancing genetic engineering and synthetic biology applications in *E. gracilis*^6^.

**Fig. 1:**
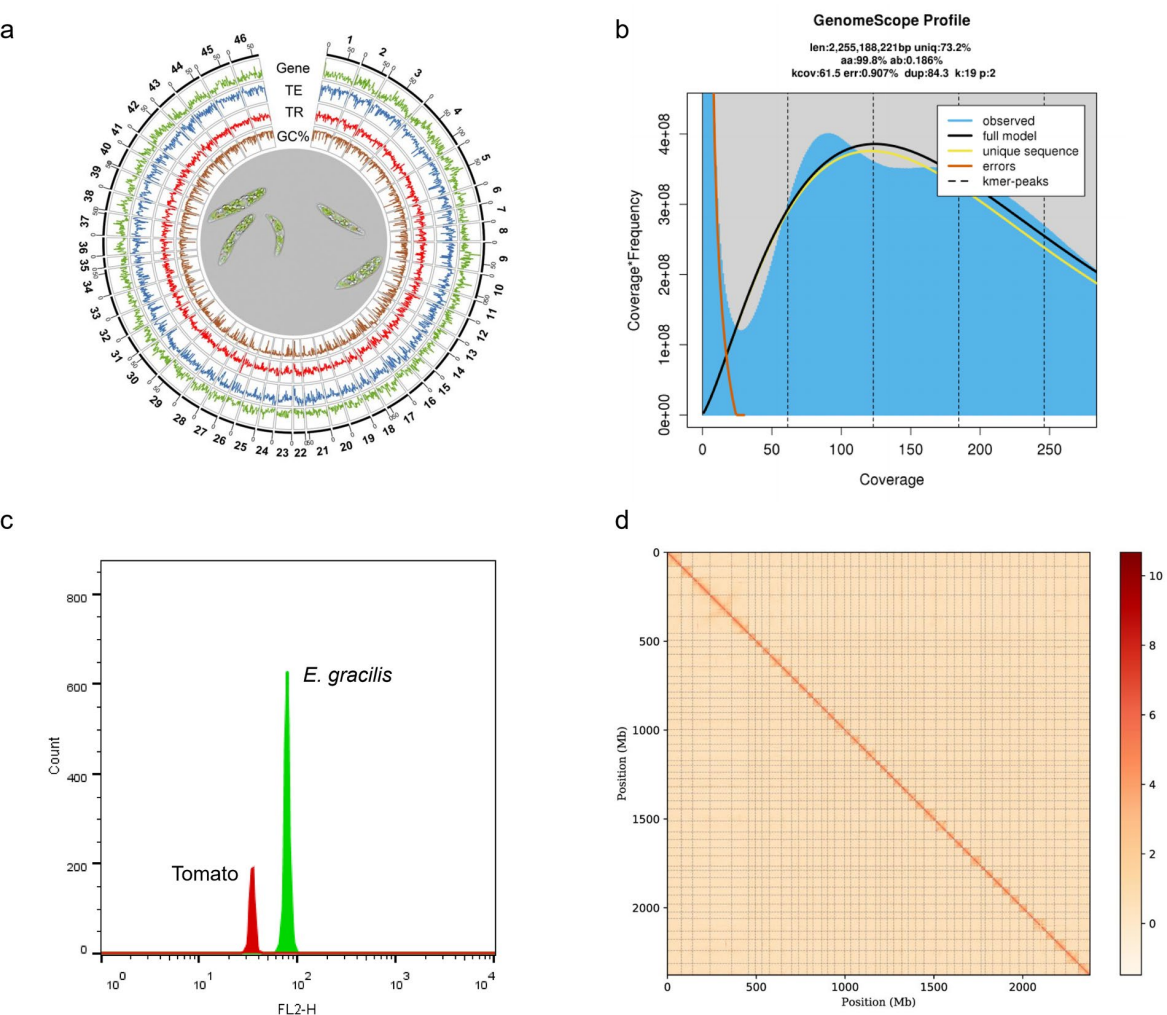
Chromosome-level assembly of the *E. gracilis* genome. (**a**) Genome landscape of the *E. gracilis*. From the outer ring to the inner ring are the distributions of chromosome length, gene density, transposable element (TE) density, tandem repeat (TR) density, and GC content, with densities calculated within a 1 Mb window. (**b**) Distribution estimation of 19-kmer. (**c**) Estimation based on flow cytometry. (**d**) Hi-C interaction heatmap illustrating the genomic interactions within the *E. gracilis* genome. The colour bar indicates contact density, ranging from red (high) to white (low).

This study addresses the existing gap by introducing a chromosome-level genome assembly of *E. gracilis* through the integration of Illumina, PacBio, Bionano, and Hi-C technologies (Table 1). The resulting assembly, spanning 2.37 Gb, with contig N50 of 619 Kb and scaffold N50 of 1.12 Mb, exhibits superior continuity (Table 2). Anchoring to 46 chromosomes (Fig. 1a) achieved a remarkable 99.83% rate, unveiling structural insights. Repetitive elements, constituting 58.84% of the genome, contribute to its complexity. The annotation of 39,362 protein-coding gene models and the assessment of 80.39% gene completeness attest to the high quality of this genome. This achievement marks a pivotal step in enhancing our comprehension of *E. gracilis*, offering a genetic foundation for both experimental and computational inquiries in this species.

**Table 1:** Statistical analysis of sequencing reads from Illumina, Pacbio, Bionano and Hi-C.

Libraries	Total Data (G)	Read length (bp)	Sequence coverage (X)
Illumina reads	264.2	150	111
PacBio reads	377.5	8563 (mean)	159
Bionano reads	306.6	—	129
Hi-C reads	402.3	150	175
Total	1350.6	—	574

**Table 2:** Assembly statistics and comparison to previous published data.

	NextDenovo	NextPolish	Sovle	3D-DNA	Ebenezer *et al*.^14^
Total length	2,184,778,864	2,183,826,505	2,378,765,506	2,374,839,547	1,435,499,417
Max length	6,602,881	6,600,390	11,664,572	121,373,930	166,587
N50	609,962	609,713	1,167,933	53,298,409	955
Total number	8,183	8,183	7,416	46	2,066,288
Average length	266,989	266,873	323,069	51,626,947	694
BUSCO score				S: 63.53%, D: 16.86%, F: 4.31%, M: 14.29%	S: 15.69%, D: 1.57%, F: 27.46%, M: 55.29%

## Methods

### Sample collection and sequencing

#### Sample preparation

The *E. gracilis* Z strain (CCAP 1224/5Z) was purchased from CCAP (Culture Collection of Algae and Protozoa, United Kingdom) and cultivated in our laboratory under autotrophic conditions using CM medium at 26 °C, with a continuous white light intensity of 80 μmol photons·m^−2^·s^−1^. Cellular samples were harvested during the mid-log phase, rapidly frozen with liquid nitrogen, and subsequently preserved at −80 °C for subsequent sequencing library preparation.

#### Library preparation and sequencing

Genomic DNA of high quality was extracted using the CTAB method. Paired-end libraries were constructed using NEBNext Ultra II DNA Library Prep Kit for Illumina (NEB, USA) and sequenced on an Illumina HiSeq2500 platform (Illumina, USA), which generated a total of 264.2 Gb Illumina data, providing approximately 111-fold coverage of the genome (Table 1). In total of 50 mg DNA were used to construct the PacBio Sequel sequencing libraries, then sequencing was performed to produce raw reads. For Bionano sequencing, high molecular weight DNA with a fragment distribution greater than 150 kb were isolated and used for DNA nicking using Nb.BssSI (NEB). The nicks were labelled and then loaded onto the Saphyr Chip nanochannel array (Bionano Genomics) and imaged using the Saphyr system and associated software (Bionano Genomics) according to the Saphyr System User Guide. The PacBio Sequel and Bionano platforms contributed 377.5 Gb and 306.6 Gb data, achieving coverages of approximately 159X and 129X, respectively (Table 1). Hi-C libraries was prepared with the standard procedure described. After digesting the genomic DNA with a restriction enzyme MboI, the sticky ends of the digested fragments were biotinylated, diluted, and then ligated to each other randomly. The prepared sequencing library was sequenced on a NovaSeq platform (Illumina, USA), which yielded a total of 402.3 Gb data with the Illumina sequencing platform (Table 1). Library preparation and sequencing of Illumina survey libraries, PacBio Sequel libraries, Bionano libraries, and all transcriptome libraries were executed by Nowbio Biotechnology Company (Yunnan, China). Frasergen Bioinformatics Co., Ltd (Wuhan, China) undertook the preparation and sequencing of Hi-C libraries on their sequencing platform.

### Genome survey and assembly

#### K-mer frequency analysis

K-mer frequencies (K = 19) were computed from filtered Illumina reads using Jellyfish^16^ (v2.2.10), serving as the basis for a genome survey conducted with GenomeScope^17^ (v2.0). The estimated genome size for *E. gracilis* was determined to be 2.25 Gb (Fig. 1b), aligning closely with the genome size estimations derived from flow cytometry analysis (2.14–2.34 Gb) (Fig. 1c).

#### Genome assembly

To assemble the genome, NextDenovo^18^ (v2.2-beta.0) was employed to generate contigs utilizing PacBio reads, followed by three rounds of Illumina read correction using NextPolish^19^ (v1.0.1). The corrected contigs underwent assembly with Bionano data using Sovle (v3.3). Subsequently, the assembled scaffolds were organized into chromosomes utilizing the 3D-DNA pipeline^20^ (v201008), followed by manual curation with JuiceBox^21^ (v2.20.00). The final assembly comprised 46 chromosomes (Fig. 1d), collectively spanning 2.37 Gb, accounting for approximately 99.83% of the entire genome assembly (Table 2), while the individual chromosome lengths ranged from 121.4 Mb (Chr4) to 22.7 Mb (Chr35) (Table 3). Comparing with the previous genome assembly^14^ of *E. gracilis* presented by Ebenezer *et al*., our assembly has much longer N50 and higher BUSCO completeness score (Table 2), which fully suggested that our result is a high-quality assembly, with superior continuity.

**Table 3:** Length of the assembled chromosome of the *E. gracilis* genome.

Chromosome No.	Length (bp)	Chromosome No.	Length (bp)
Chromosome 1	78,328,259	Chromosome 24	36,335,361
Chromosome 2	63,227,137	Chromosome 25	45,701,548
Chromosome 3	97,963,227	Chromosome 26	36,011,715
Chromosome 4	121,373,930	Chromosome 27	41,894,679
Chromosome 5	94,835,252	Chromosome 28	46,635,366
Chromosome 6	38,199,897	Chromosome 29	67,424,199
Chromosome 7	36,901,641	Chromosome 30	61,758,312
Chromosome 8	42,062,667	Chromosome 31	42,006,156
Chromosome 9	68,447,031	Chromosome 32	46,401,773
Chromosome 10	56,373,607	Chromosome 33	44,833,052
Chromosome 11	39,739,124	Chromosome 34	58,733,468
Chromosome 12	47,646,790	Chromosome 35	22,709,709
Chromosome 13	33,780,947	Chromosome 36	41,866,910
Chromosome 14	47,789,021	Chromosome 37	53,298,409
Chromosome 15	33,529,397	Chromosome 38	44,312,966
Chromosome 16	39,053,207	Chromosome 39	50,770,958
Chromosome 17	56,341,894	Chromosome 40	44,907,322
Chromosome 18	34,489,017	Chromosome 41	35,730,237
Chromosome 19	47,523,710	Chromosome 42	71,665,632
Chromosome 20	38,438,652	Chromosome 43	46,340,383
Chromosome 21	54,477,733	Chromosome 44	63,186,876
Chromosome 22	26,325,358	Chromosome 45	72,099,000
Chromosome 23	38,767,632	Chromosome 46	64,600,386

### Genome repeat and ncRNA analysis

#### Repeat sequence prediction

A hybrid approach, incorporating both *ab initio* and homology-based methodologies, was employed to predict repeat sequences within the genome. For *ab initio* prediction, LTR_FINDER^22^ (v1.07) and ltrharvest^23^ (v1.5.10) were used to predict LTR retrotransposons, and the results were integrated using LTR_retriever^24^ (v2.8). Meanwhile, RepeatModeler^25^ (v2.0) was also used to identify repeats. Then the results of LTR_retriever and RepeatModeler were merged as a custom library and fed to Repeatmasker^26^ (v.4.0.9) to predict TEs. Simultaneously, homology-based annotation employed RepeatMasker^26^ (v.4.0.9) and RepeatProteinMask^26^ (v.4.0.9) against Repbase^27^ (Release 20181026). TRF^28^ (v4.0.9) was used for searching tandem repeats. Following redundancy elimination, a total of 1.4 Gb of repeat sequences were identified, constituting 58.84% of the *E. gracilis* genome. The repeat sequences predicted by TRF, Repeatmasker, Proteinmask and *ab initio* pipeline covered 9.85%, 1.89%, 2.07% and 52.75% of the genome sequence, respectively. Within the repeat elements, 32.73% remained unclassified, while long terminal repeats (LTRs) represented 32.81% of the genome. DNA elements, long interspersed nuclear elements (LINEs), and short interspersed nuclear elements (SINEs) accounted for 4.60%, 1.49%, and 0.11% of the genome, respectively (Table 4).

**Table 4:** Classification of the TE sequences in the *E. gracilis* genome.

Type	Repbase TEs		TE protiens		*Ab initio*		Combined TEs	
	Length (bp)	% in genome	Length (bp)	% in genome	Length (bp)	% in genome	Length (bp)	% in genome
DNA	37,348,003	1.57	242,510	0.01	71,973,983	3.026	109,510,738	4.604
LINE	3,053,732	0.128	7,121,290	0.299	25,168,911	1.058	35,321,327	1.485
SINE	1,457	0	0	0	2,611,069	0.11	2,612,472	0.11
LTR	7,751,603	0.326	41,909,336	1.762	470,824,413	19.793	518,870,377	21.813
Unknown	16,431	0.001	1,536	0	778,461,146	32.725	778,478,966	32.726
Total	44,845,691	1.885	49,272,342	2.071	1,225,074,726	51.5	1,316,869,099	55.35

#### Noncoding RNA annotation

To annotate noncoding RNA (ncRNA), tRNAScan-SE^29^ (v1.3.1) and blast^30^ (v2.2.26) were applied for tRNA and rRNA prediction, respectively. Additionally, Rfam^31^ (v9.1) and INFERNAL^32^ (v0.81) were utilized for miRNA and snRNA prediction on the genome. This comprehensive approach identified four types of ncRNAs within the *E. gracilis* genome, encompassing 188 miRNAs, 4882 tRNAs, 223 rRNAs, and 165 snRNAs.

### Gene prediction and annotation

#### Pre-processing and *de novo* assembly

The Illumina RNA-seq data underwent initial filtration utilizing Trimmomatic^33^ (v0.32) to obtain clean reads, subsequently employed in Trinity^34^ (v2.1.1) for *de novo* assembly. The Pacbio full-length RNA-seq dataset was refined to derive consensus sequences using smrtlink (v6.0.0).

#### Transcript integration and *ab initio* prediction

The two distinct sets of transcripts were amalgamated via PASA^35^ (v2.4.1) for *ab initio* gene prediction, utilizing Augustus^36^ (v2.5.5) and SNAP^37^ (2006-07-28). Homology annotation was conducted with ten representative species, including *Bodo saltans*, *Naegleria gruberi*, *Phytomonas sp*., *Chlamydomonas reinhardtii*, *Leishmania major Friedlin*, *Nannochloropsis gaditana*, *Trypanosoma brucei*, *Cyanidioschyzon merolae*, *Leptomonas pyrrhocoris*, and *Perkinsela sp*., downloaded from NCBI. The comprehensive integration of all data and generation of the predicted gene set were accomplished using MAKER^38^ (v3.01.02). The ensuing analysis revealed a total of 32,806 genes and 39,362 coding DNA sequences (CDSs) within the *E. gracilis* genome, with an average CDS length of 1,149 bp and an average of 8 exons per gene.

#### Functional annotation

For functional annotation, blastp^30^ (v2.2.26) was applied to align protein-coding genes with KEGG^39^ database. The GO Ontology^40^ (GO) and InterPro^41^ function were obtained using InterProScan. The subsequent functional annotation of CDSs demonstrated coverage of 28.2%, 40.6%, and 50.2% across the GO, InterPro, and KEGG databases, respectively, with a cumulative 57.3% of CDSs annotated in at least one database.

## Data Records

### Sequencing data deposit

The comprehensive *E. gracilis* genome project has been archived in the Genome Sequence Archive^42,43^ (GSA) under the accession^44^ CRA013190, except that the Illumina RNA-seq data have been archived in the SRA at NCBI SRP353774^45^.

### Assembly deposit

The assembly of the *E. gracilis* genome, along with its corresponding annotation file, is available at figshare^46^ and NCBI GenBank with accession number GCA_039621445.1^47^.

## Technical Validation

### Genome assembly quality assessment

The quality assessment of the *E. gracilis* genome assembly was executed through two distinct methodologies. Firstly, the completeness of the assembly was rigorously validated utilizing compleasm^48^ (v0.2.2), an improved BUSCO^49^ workflow based on miniprot, with specific parameters (-m lite–min_identity 0.8–min_length_percent 0.9–min_rise 0.9), and employing the eukaryota_odb10 (v5, 2020-09-10) reference gene set (n = 255). The final BUSCO analysis yielded a completeness score of 80.39%, comprised of 162 (63.53%) single-copy BUSCOs, 43 (16.86%) duplicated BUSCOs, 11 (4.31%) fragmented BUSCOs, and 39 (15.29%) missing BUSCOs. Secondly, to affirm the accuracy and integrity of the genome survey, the filtered Illumina short reads utilized were aligned back to the *E. gracilis* genome utilizing the Burrows-Wheeler aligner^50^ (BWA, v0.7.17-r1188). This meticulous alignment process revealed an impressive mapping rate of 99.42% for the short reads against the genome. The combination of these validated results attests to the high-quality nature of the *E. gracilis* genome assembly.

## Data Availability

All commands and pipelines employed for data processing adhered strictly to the guidelines specified in the manuals of the pertinent bioinformatics software, with the parameters explicitly detailed in the Methods section. In instances where no specific parameters were explicitly stated for a particular software, default parameters were applied. It is noteworthy that no bespoke scripts or custom code were formulated or utilized throughout the course of this study.
